# Emerging Concepts in the Surgical Management of Peri-Acetabular Metastatic Bone Disease

**DOI:** 10.3390/curroncol28040238

**Published:** 2021-07-17

**Authors:** Aaron Gazendam, Daniel Axelrod, David Wilson, Michelle Ghert

**Affiliations:** Division of Orthopaedic Surgery, Department of Surgery, McMaster University, Hamilton, ON L8V 1C3, Canada; daniel.axelrod@medportal.ca (D.A.); wilsondaj@gmail.com (D.W.); ghertm@mcmaster.ca (M.G.)

**Keywords:** peri-acetabular metastases, cementoplasty, Harrington procedure, acetabular metastases

## Abstract

The pelvis is a common site of metastatic bone disease. Peri-acetabular lesions are particularly challenging and can cause severe pain, disability and pathologic fractures. Surgical management of these lesions has historically consisted of cementoplasty for contained lesions and Harrington reconstructions for larger, more destructive lesions. Due to the limitations of these procedures, a number of novel procedures have been developed to manage this challenging problem. Percutaneous techniques—including acetabular screw fixation and cementoplasty augmented with screws—have been developed to minimize surgical morbidity. Recent literature has demonstrated a reliable reduction in pain and improvement in function in appropriately selected patients. Several adjuncts to the Harrington procedure have been utilized in recent years to reduce complication rates. The use of constrained liners and dual mobility bearings have reduced the historically high dislocation rates. Cage constructs and porous tantalum implants are becoming increasingly common in the management of large bony defects and destructive lesions. With novel and evolving surgical techniques, surgeons are presented with a variety of surgical options to manage this challenging condition. Physicians must take into account the patients’ overall health status, oncologic prognosis and anatomic location and extent of disease when developing an appropriate surgical plan.

## 1. Introduction

With advances in systematic therapies alongside an aging population, the number of patients living with metastatic cancer is increasing [1]. Improved oncologic care has to led to longer survival in patients with metastatic disease and may exceed 5 years in patients with prostate and breast carcinoma or multiple myeloma [2,3,4]. Bone is the third most common site of metastasis after the lung and liver with the incidence varying based on primary tumor type [5,6]. Metastatic bone disease (MBD) is a major contributor to morbidity in cancer patients and can lead to pain, reduced quality of life, pathologic fractures, hypercalcemia and anemia [5]. 

Pelvic metastases represent the third most common site of MBD, accounting for 10–20% of metastatic bone lesions [7,8,9]. Due to their anatomic location supporting the hip joint, peri-acetabular lesions are particularly challenging and can cause severe pain, disability and pathologic fractures [10]. As a weightbearing joint, progressive peri-acetabular lesions can compromise the mechanical stability of the pelvic ring. Nonoperative treatment includes protected weightbearing, analgesics, bone modifying agents such as bisphosphonates, radiation and systematic therapies. Surgical management is indicated if symptoms are intolerable despite nonoperative interventions, or with impending catastrophic fracture or collapse of the pelvis [9]. Importantly, the patient’s expected longevity must be longer than the surgical recovery to allow for a net improvement in quality of life.

The Harrington classification is the most commonly used classification system when describing peri-acetabular metastatic lesions [11]. There are four groups in the original Harrington classification (Table 1). Group I represents those with an intact subchondral bone of the acetabulum. Group II is defined as lesions that have destroyed the medial wall but demonstrate an intact acetabular roof and lateral wall. Group III is defined as destruction of the medial wall, superior aspect (roof), and lateral rim of the acetabulum. Group IV is defined as patients with solitary metastases that can be resected en bloc with anticipation of a cure.

**Figure 1:**
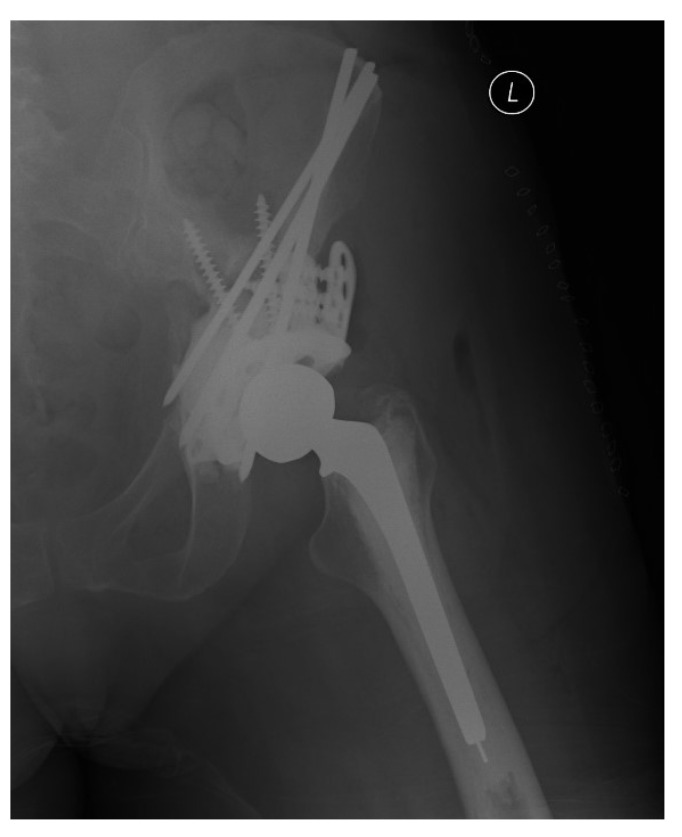
Traditional Harrington Procedure with superior titanium acetabular augmentation.

In 1981, Harrington described a surgical technique that involved placing threaded Steinmann pins and cement to reconstruct acetabular defects to augment a cemented total hip arthroplasty in the setting of MBD (Figure 1) [11]. The goal was to allow transmission of weight-bearing forces to intact bone in the pelvis. The core principles of management of MBD described by Harrington remain true today. Modern surgical approaches and techniques are largely dictated by the size and location of the defects created by the metastases. Extensive defects are still most commonly treated with modified Harrington reconstructions using a combination of antegrade or retrograde pins or screws with cementation combined with a total hip arthroplasty [12]. Despite reliable improvements in pain and function, these open procedures are plagued by extensive blood loss and large surgical wounds. 

**Figure 2:**
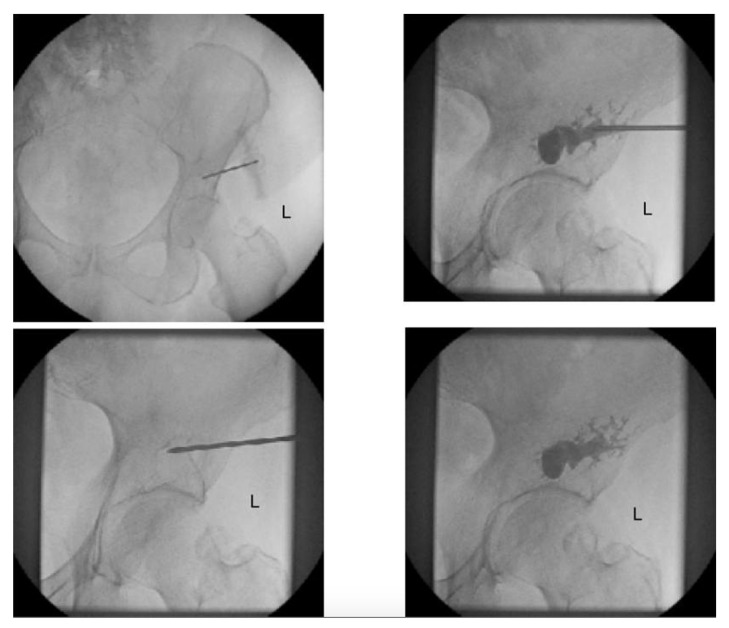
Intraoperative fluoroscopy demonstrating percutaneous cementoplasty of a lytic metastatic lesion in the left acetabulum, courtesy of Harris et al. [18].

Smaller and contained defects have been historically treated nonoperatively or with cementoplasty [13]. Cementoplasty involves the injection of cement into osteolytic lesions to provide stability and pain relief. Newer techniques allow for percutaneous, minimally invasive, image-guided cementoplasty, reducing the risks of complications (Figure 2) [14]. These techniques have been shown to achieve excellent short-term results with immediate stability and improvements in pain control [15]. This technique is not appropriate for larger, more destructive lesions in which the structural integrity of the acetabulum is compromised [16]. Additionally, cementoplasty may not provide adequate longer-term relief—and the risk of future pathologic fracture persists [15,17].

## 2. Novel Techniques

### 2.1. Percutaneous Screw Fixation

The utilization of percutaneous techniques for acetabular column fractures was introduced in the 1990s and has been widely adopted in the trauma literature [19]. In the trauma setting, fixation of the anterior and posterior acetabular columns independently allows for immediate stabilization of the acetabulum and early weightbearing [20,21]. Percutaneous fixation affords a number of advantages over open reduction and internal fixation, including minimal soft tissue disruption, reduced blood loss and shorter operative time [22]. The major limitation of percutaneous techniques is the reduced ability to achieve an anatomic reduction.

More recently, these techniques have been expanded to treat patients with painful peri-acetabular metastases. Yang et al. described a tripod configuration with percutaneous screw placement in the anterior column, posterior column and a 3rd trans-columnar screw [23]. The authors utilized 6.5–8.0 mm fully cannulated screws, placed under fluoroscopic guidance, and demonstrated significant improvements in VAS pain and functional outcomes in 20 patients with Harrington class-II and III lesions at 3 months follow-up. They reported no intraoperative and perioperative complications related to the procedure. When conversion to THA was indicated due to disease progression, it was uncomplicated, and the acetabular screws were utilized as rebars to support a cemented acetabular cup. 

However, there are a number of limitations to note when considering percutaneous fixation of peri-acetabular metastases. Firstly, percutaneous fixation relies on an intimate knowledge of the pelvic anatomy and intraoperative fluoroscopic views and may not be feasible for the majority of non-trauma-trained surgeons. Secondly, in lesions with a significant amount of destruction or rapid progression, screw fixation may not be sufficient. It is also difficult to obtain an adequate reduction in displaced fractures with percutaneous techniques. Finally, the hardware utilized for fixation are generally included in specialized trauma sets and may not be readily available at all institutions. 

### 2.2. Percutaneous Cementoplasty Adjuncts 

**Figure 3:**
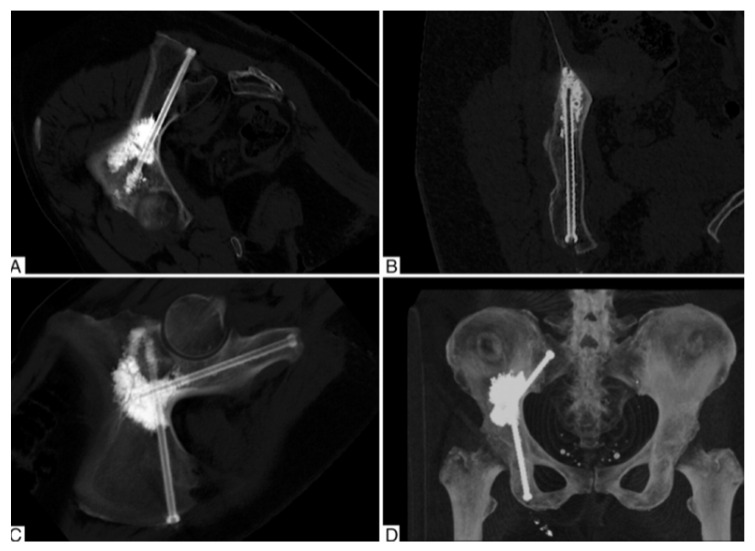
Computed tomography cuts (**A**–**C**) and 3D rendering (**D**) of transischiatic cementoplasty with percutaneous acetabular screw fixation. Image courtesy of Roux et al. [24].

In acknowledgement of the limitations of cementoplasty, several potential augments have been introduced. An evolving approach involves a combination of cementoplasty and percutaneous screw fixation in a rebar-type fashion. The addition of screws allows offers a more stable construct, particularly with sheer, rotational or distracting forces. Roux et al. reported a single center case series of 100 patients with peri-acetabular metastases with percutaneous image-guided cementoplasty with acetabular column screw augmentation (Figure 3) [24]. Patients experienced significant pain relief and reduction in opioid consumption postoperatively. They demonstrated a low complication rate and 5% reintervention rate due to secondary pathologic fractures. Cement deposition enhances the torsional stability of the screws and has the potential to reduce hardware loosening postoperatively [25]. For smaller defects, cementation can be performed utilizing a trocar placed through cannulated screws [26]. For larger defects, cementation can be placed after screw fixation using separate percutaneous access points [26]. Similar to screw fixation alone, there are limitations to percutaneous-only techniques for fixation. 

**Figure 4:**
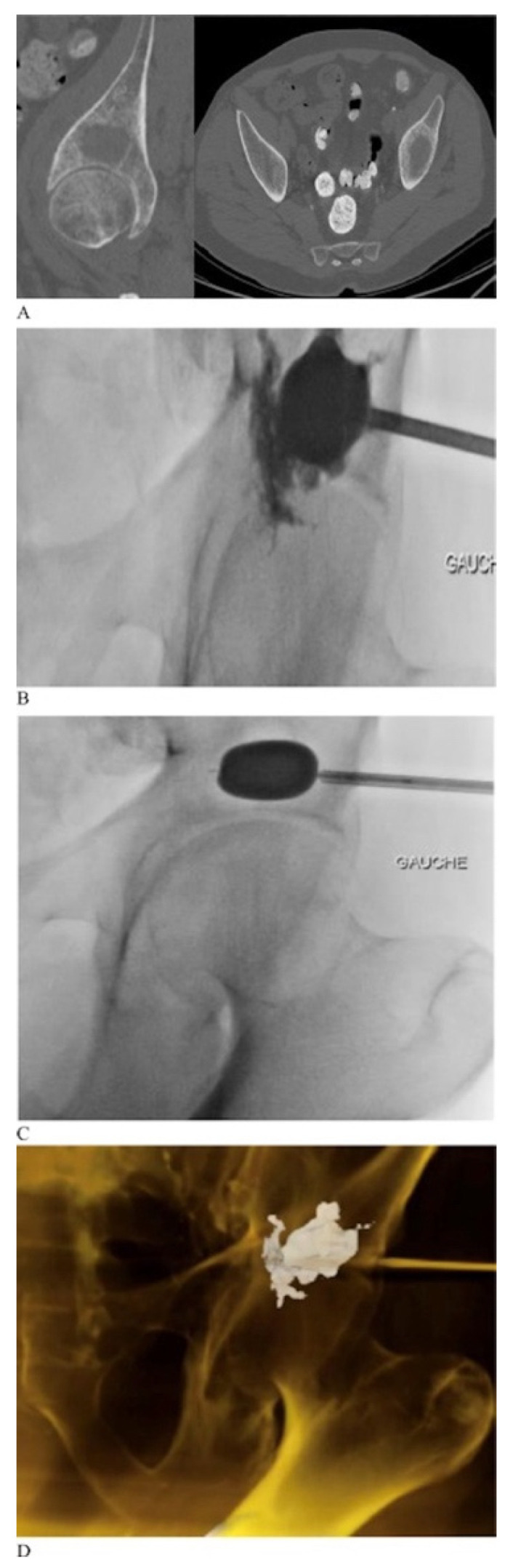
A patient with left superior acetabular metastatic lesion demonstrated on axial and coronal cuts of a preoperative computed tomography scan (**A**). The patient underwent balloon augmented cementoplasty (**B**–**D**). Images borrowed from Couraud et al. (2018) [17].

Other adjuncts to intraosseous cementoplasty have been introduced in efforts to improve its efficacy [14]. Given that incomplete cement filling is associated with fracture progression, techniques have been developed to maximize cement filling [27]. Kurup et al. employed cementoplasty augmentation balloons traditionally used in kyphoplasty to maximize cement filling and minimize cement spillage in peri-acetabular lesions [28]. They demonstrated that it was a feasible and safe technique with excellent cement filling and minimal cement spillage. Similar results were found by Couraud et al. who demonstrated a reduction in pain and improvement in quality of life utilizing this technique (Figure 4) [17]. Cementoplasty can also be combined with percutaneous radiofrequency ablation and can aid in tumor destruction and increase the cement filling rate [14,29]. Lee et al. recently reported on a combined technique with percutaneous ablation, osteoplasty and internal screw fixation [30]. Short term results at 2-week follow-up demonstrated statistically significant improvements in VAS pain scores and MSTS functional outcomes with low complications rates.

### 2.3. Limitations of Percutaneous Techniques

It is clear that there is a growing body of literature that demonstrates several advantages of percutaneous treatment of peri-acetabular metastases. Minimally invasive techniques improve pain and functional outcomes while minimizing surgical morbidity and healthcare costs. However, a major limitation exists: percutaneous techniques do not allow for local disease control. Without a formal open curettage, local disease remains and has the potential to progress. This is most relevant for primary malignancies that are less responsive to systemic and radiation therapies. Given the lack of comparative trials, it remains unclear if a formal curettage and debulking procedure has an important effect on disease progression, complications and overall survival. Additionally, many patients present with advanced collapse and destruction with acetabular protrusion. These lesions are not amenable to percutaneous fixation and open reconstruction may be more appropriate in these cases. 

### 2.4. Harrington Procedure Adjuncts 

Historically, the Harrington procedure was plagued by high rates of dislocation and aseptic failures [31]. In addition to advances in percutaneous techniques, open reconstructive options have evolved since the first description by Harrington. Constrained liners—popularized in arthroplasty to manage recurrent instability—have been utilized as an adjunct in modified Harrington techniques. Bagsby et al. demonstrated no cases of dislocation or component failure in 47 patients who underwent a modified Harrington procedure with constrained liners [31]. Dual mobility bearings have increased in popularity as they have been shown to reduce dislocation rates without the risk of aseptic loosening found in constrained liners [32]. Wegrzyn et al. reported a case series of 126 patients with peri-acetabular metastatic disease treated with a Harrington procedure and dual mobility bearings [33]. They demonstrated a dislocation rate of 2% at a mean follow-up of 33 months. 

**Figure 5:**
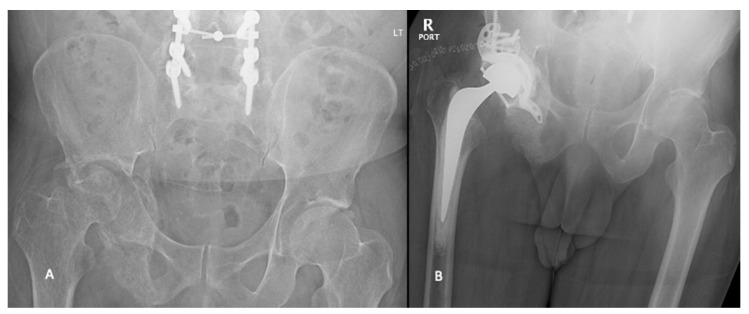
Pre- (**A**) and postoperative (**B**) radiographs of patient with metastatic small cell lung cancer who underwent cemented total hip arthroplasty with cage construct.

Similarly, the advent of antiprotrusio acetabular cages has allowed surgeons to address the large pelvic defects often found in Harrington III lesions (Figure 5). Acetabular cages have been used both with and without retrograde screw or Steinman pin fixation and with ischial fixation and cementation [34,35,36]. Tsagozis et al. reported on a case series of 70 patients undergoing a modified Harrington procedure with antiprotrusio cage with retrograde screw fixation. They demonstrated 89% prosthesis survival at 5 years but did note a high dislocation rate of 18.5% [34]. Plummer et al. utilized a similar technique with the addition of dual mobility components in a small series of patients and reported no dislocations at 2 years [37]. Rowell et al. reported on 46 patients treated with cementation and an acetabular cage with fixation into the ischium and ilium. They demonstrated excellent return to function with an overall reoperation rate of only 16% at 4 years [36]. 

### 2.5. Porous Tantalum Implants 

Porous tantalum implants have been readily used in revision hip arthroplasty to address large acetabular defects [38]. They have several unique properties—including a high friction surface, high porosity and low modulus of elasticity—that render them conducive to biologic fixation [38]. Recent evidence has suggested that highly porous tantalum implants with augments or cage constructions are a durable alternative to Harrington-type reconstructions [39,40]. Houdek et al. retrospectively compared 115 patients who underwent either a cemented Harrington technique or tantalum acetabular reconstruction [39]. They concluded that both groups achieved significant functional improvements postoperatively and the tantalum reconstruction group had a lower 10-year cumulative incidence of revision (9% vs. 0%, *p* = 0.09). Uncemented constructs rely on biologic fixation and may be valuable in populations with a prolonged life expectancy but would not be considered viable options for patients who are likely to undergo adjuvant local irradiation. Otherwise, cemented components can be as reliable and offer immediate fixation.

### 2.6. Endoprosthetic Reconstructions

Periacetabular endoprostheses are commonly used in reconstructive procedures following primary bone tumor resection [41]. Historically, endoprosthetic reconstructions have been limited by high complication rates. However, modern advances have improved functional outcomes and reduced complications in both primary and metastatic bone tumor populations [42,43]. Some authors have advocated for the use of pelvic endoprostheses in Group III Harrington lesions with extensive bone loss. Wei et al. utilized a modular hemipelvic endoprosthesis in patients with massive bone loss and demonstrated reduced intraoperative blood loss, improved functional outcomes and improved recurrence-free survival compared to patients treated with traditional Harrington reconstructions [43]. Complication rates remain high with endoprosthetic reconstruction; however, this is likely in part due to the destructive nature of the lesions for which they are utilized [44].

## 3. Conclusions

As novel oncologic therapies have allowed patients to live longer with cancer, the number of patients with MBD will continue to rise. Peri-acetabular metastases represent a spectrum of disease that can be debilitating and greatly impact patient’s quality of life. Surgical management of peri-acetabular MBD has the potential to provide rapid improvements in pain and function. With novel and evolving surgical techniques, surgeons are presented with a variety of surgical options to manage this challenging condition (Table 2). Physicians must take into account the patients’ overall health status, oncologic prognosis and anatomic location and extent of the disease when considering the optimal surgical approach. Endoprostheses are a valuable tool and should remain in the armamentarium for the management of massive peri-acetabular bone loss secondary to MBD.

## Figures and Tables

**Table 1:** Harrington classification describing peri-acetabular metastatic bone disease.

Group	Description
I	Lateral cortices and superior/medial walls intact
II	Deficient medial wall
III	Acetabular dome defect
IV	Isolated lesion that could be resected with curative intent

**Table 2:** Surgical options for management of peri-acetabular metastatic bone disease.

Surgical Procedures	Utility	Drawbacks
Cementoplasty	Minimally invasive Immediate stability Reliable improvements in pain	Inadequate for large defects Cement extravasation Short-term relief
Percutaneous Screw Fixation	Minimally invasive Reliable improvements in pain	Does not decrease tumor burden Technically challenging Pathologic fractures not easily reduced
Percutaneous Screws + Cementoplasty	More stability than either construct alone Minimally invasive	Inadequate for large defects Does not decrease tumor burden
Harrington Procedure	Stable construct Dual mobility liners have improved dislocation rates	High surgical morbidity Historically high rates of aseptic failure and dislocation
Acetabular Cages	Stability in large defects Can be combined with Harrington rods	High surgical morbidity High rates of aseptic loosening
Porous Tantalum Implants	Conducive to biologic fixation Durable	Relies on biologic fixation
Endoprosthetic Reconstructions	Addresses massive bony defects Modular or custom	High surgical morbidity High complication rates
